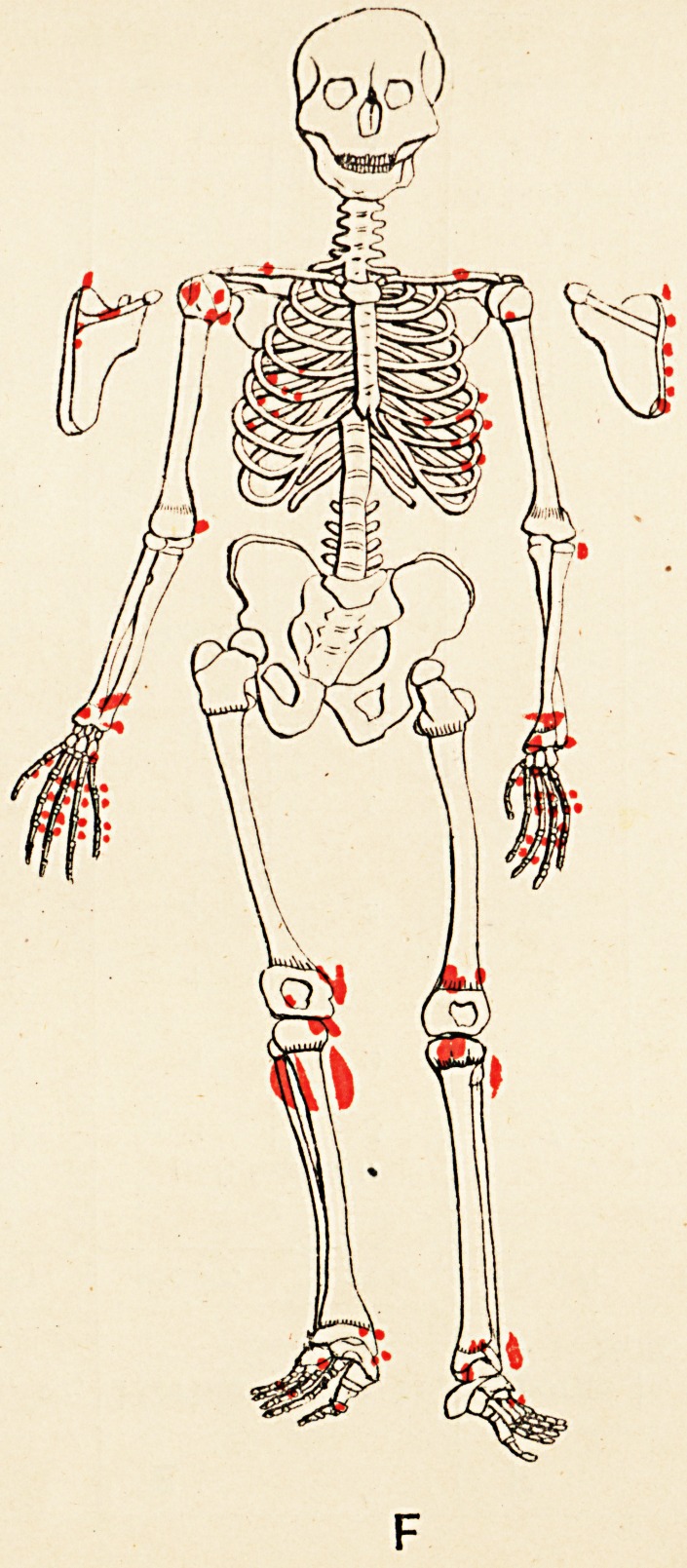# Multiple Cancellous Exostoses

**Published:** 1890-12

**Authors:** Ewen J. Maclean

**Affiliations:** House-Surgeon Bristol Hospital for Sick Children and Women


THE BRISTOL

Medico-Chirurgical Journal.

DECEMBER, 1890.

### MULTIPLE CANCELLOUS EXOSTOSES.

BY

Ewen J. Maclean, M.B. & C.M. (Edin.),

*House-Surgeon Bristol Hospital for Sick Children and Women.*

The existence of multiple exostoses, though by no means rare, is seldom so well marked as in the following series of cases occurring in the same family.

As causative of or coincident with this abnormality, various factors in the family or personal history have been cited by different writers. Amongst these, *heredity* takes a prominent position. This is well seen in these cases, where the children have clearly inherited the exostoses from the father, whose sister and two brothers are similarly affected. Unfortunately, no reliable statement can be obtained as to their occurrence or otherwise in the grandfather or grandmother. The only fact worth noting with regard to them is, that between these two there was some 35 years' disparity of age, the man being the elder.

*Syphilis, rheumatism, inter-marriage,* have been noted as concomitants; but no history of these is present in the family. Needless to say, the obtainable data alike afford no support to the *retrograde metamorphosis theory,* which is largely founded on the fact that the sites of these exostoses correspond to an interesting degree to those of the ossified tendons in birds and of some bony projections normally present in certain of the lower animals.

Some evidences of *rickets* are seen. The accompanying skeletal diagrams indicate the positions and, as far as possible, the forms of the exostoses. The conditions of the aunt, father, daughter, and three sons are represented respectively. The accompanying engraving from a photograph (kindly taken by Mr. John Griffiths) shows the most prominent tumours in the boys.

Mr. Norton has courteously allowed the case of the youngest boy to be described, who was under his care in the hospital. An operation on this boy and its results are referred to in the text following.

Reference to the diagrams shows, amongst others, the following points (each scapula is seen in post-view by its corresponding side) :—

(1) That the exostoses do not all originate from the neighbourhood of epiphyses.

(2) That in every instance they do originate thus, or from the usual sites of muscular attachment, or from both these positions.

(3) That the bones of the face and spinal column (to palpation), the sternum and patellæ, are exempted, and the bones of the head almost so; whilst the long bones, scapulæ, and ribs are extensively affected.

(4) That the pelvis (ilium) is affected in three of the six cases (B, D, and E).

(5) That sesamoid bones may exist concurrently with the exostoses (A and C).

(6) That the exostotic growth may bind together two adjacent long bones, *e.g.* the tibia and fibula of right limb of Case F; also lower ends of left tibia and fibula in Case E.

(7) That no exostoses of the subungual variety are present.

(8) That the disposition of the growths is to a high degree symmetrical.

The mother deems it quite possible the "bones" were present at birth, although their presence was not noticed till some weeks or months thereafter. In the father's case the tumours increased during childhood and until he was about 20 years of age, when they apparently ceased to grow, and, he thinks, since have somewhat diminished in size.

No inconvenience from their presence is said to be felt, excepting on the score of their position round the ankles. This interferes with the proper lacing of the boots, and, at times, the tissues superjacent to the exostoses in this situation become tender and somewhat inflamed, evidently from the undue friction between them and the boot during progression. Bursæ may be developed. The method of locomotion is normal, excepting in the case of the youngest boy, who has an enormous exostosis, about the size of a very large orange to palpation (see photograph, side view), springing from the posterior surfaces of the heads and from the upper third of posterior surfaces of shafts of right tibia and fibula. Little else seems to intervene between the palpating hand and the summit of the bony tumour besides skin and some probable subcutaneous fat; no muscular contraction is felt over it during pedal extension, and no displaced arteries or nerves are detected. The gait of this boy is somewhat "rolling," from side to side, and is rather of the nautical type.

The structure of the exostosis removed by operation (see *infra*) was of the usual cancellous character, easily cut, amply porous on section, and capped by a thin coating of cartilage.

With regard to the treatment, it may be of interest to record that Case F was admitted into the hospital for the removal of an inconveniently prominent exostosis on the left external malleolus. The removal was readily effected by Mr. Norton; and when examined on the sixth day after operation, firm primary union of the wound had occurred. But even in this short time three small accessory exostoses had developed around the site of the base of the one removed, and there was general thickening of the lower end of the fibula. In the course of some weeks this latter became less marked. The father was also operated on at the Infirmary, some years ago, with similar results.

The attached table indicates for the several cases the number of exostoses and the bones from which they grow. From it we may deduce, with regard to these six cases, that in the numerical order of affection of the bones the tibia and scapula stand highest; that of the long bones, next to the tibia comes the humerus; then the femur and fibula, followed at some distance by the radius; then the ulna: so that in the upper extremity, the humerus is more affected than the radius, and the radius than the ulna; whilst in the lower extremity, the tibia is more affected than femur or fibula, the two latter being about equal.

A

B

C

D

E

F

### TABLE.

| CASE ... ... | | A | | B | | C | | D | | E | | F | |
|---|---|---|---|---|---|---|---|---|---|---|---|---|---|
| NAMES OF BONES. | | Sarah J., æt. 53, paternal Aunt. | | Joseph J., æt. 43, Father. | | Ellen J., æt. 16, Daughter. | | Walter J., æt. 12, Son. | | Herbert J., æt. 10, Son. | | George J., æt. 8, Son. | |
| No. | | RIGHT. | LEFT. | RIGHT. | LEFT. | RIGHT. | LEFT. | RIGHT. | LEFT. | RIGHT. | LEFT. | RIGHT. | LEFT. |
| 3 | Cranium ... ... | – | | 1 (over inion). | | – | | 1 (over inion). | | 1 (left frontal). | | – | |
| 33 | Ribs (on bodies) .. | – | – | – | – | – | – | 7 | 5 | 3 | 3 | 7 | 8 |
| 19 | Clavicle ... ... | – | – | 3 | 3 | 2 | 2 | 1 | 1 | 3 | 2 | 1 | 1 |
| 50 | Scapula ... ... | – | – | 1 | 1 | 6 | 3 | 6 | 7 | 4 | 11 | 5 | 6 |
| 34 | Humerus ... | 1 (very prominent). | – | 3 | 4 | 3 | 3 | 6 | 3 | 4 | 2 | 4 | 1 |
| 18 | Radius ... ... | 2 | 2 | 2 | 1 | 1 | 1 | 2 | 1 | 1 | 1 | 2 | 2 |
| 13 | Ulna ... ... | 1 | 1 | 1 | 1 | 1 | 1 | 1 | – | 2 | 1 | 1 | 2 |
| 127 | Metacarpals and Phalanges | 10 | 6 | 5 | 2 | 11 | 18 | 14 | 12 | 10 | 9 | 14 | 16 |
| 8 | Os innominatum .. | ? | ? | 1 (ilium) | 1 (ilium) | – | – | 1 (ilium) | 3 (ilium) | – | 2 (ilium) | – | – |
| 28 | Femur ... ... | – | 1 | 3 | 2 | 1 | 1 | 5 | 5 | 2 | 2 | 3 | 3 |
| 51 | Tibia ... ... | 3 | 2 | 4 | 6 | 6 | 3 | 4 | 4 | 5 | 3 | 5 | 6 |
| 27 | Fibula ... ... | 1 | 1 | 3 | 2 | 1 | 2 | 2 | 4 | 3 | 2 | 1 | 5 |
| 1 | Tarsus ... ... | – | – | – | – | – | – | – | – | – | – | 1 on scaphoid tubercle | – |
| 42 | Metatarsals and Phalanges | – | – | 2 | – | 6 | 7 | 8 | 5 | 3 | 4 | 5 | 2 |
| | Individual Totals = | 31 | | 52 | | 79 | | 108 | | 83 | | 101 | |

Total exostoses in six cases 454.

Again: that, excluding the hand-bones and scapula from the upper extremity, and the foot-bones with os innominatum from the lower, the latter is almost twice as much affected; but that apart from these exclusions, the order is more than reversed.

It will be seen from the diagrams that the growth is more exuberant at those ends of the long bones whose epiphyses unite later with the diaphyses, and less exuberant at those ends towards which the nutrient arteries are directed.

It is to be noted that in the case of metacarpals, metatarsals, and phalanges, by far the majority of exostoses are at the proximal and distal ends of these bones, and mainly on the dorsal aspect. A few, however, occur in the course of their shafts. Those near joints may cause marked distortion of a finger or toe, as in cases B and D.

There is outward-convexity curvature (supine position) of the left forearms of A and B, and of right forearm of D. In B, the whole of left arm is comparatively small, and the power of pronation and supination is lost, the radius and ulna being fused or bound together. (See diagram.) He attributes the condition to a wrench of the limb, during heavy work, when not 20 years of age.

This curvature (convex outwards) of *left* forearm has been not unfrequently recorded in conjunction with multiple exostoses: amongst others, by Jones, of Manchester Children's Hospital, in 1878, and later by Bruce Clark and Haslam, of Birmingham, in 1887. It has been attributed to overgrowth of the lower end of the radius, or defective development of that of the ulna.

The photograph has reference to the cases D, E, and F respectively, and exhibits the only visibly appreciable tumours of the group.

## Figures and Tables

**Figure f1:**
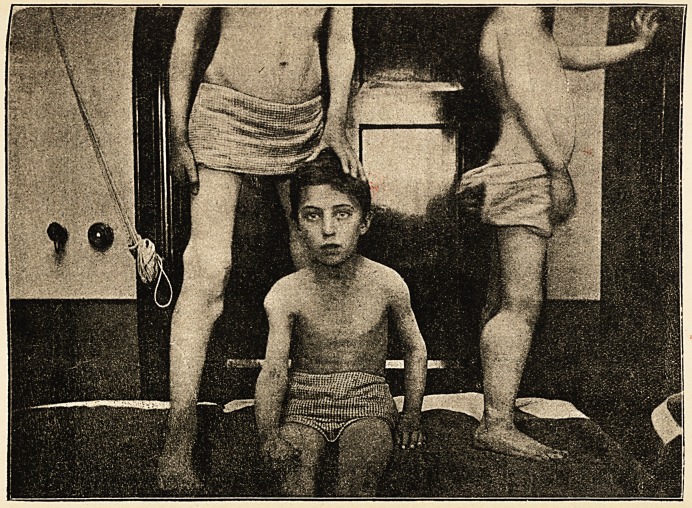


**A f2:**
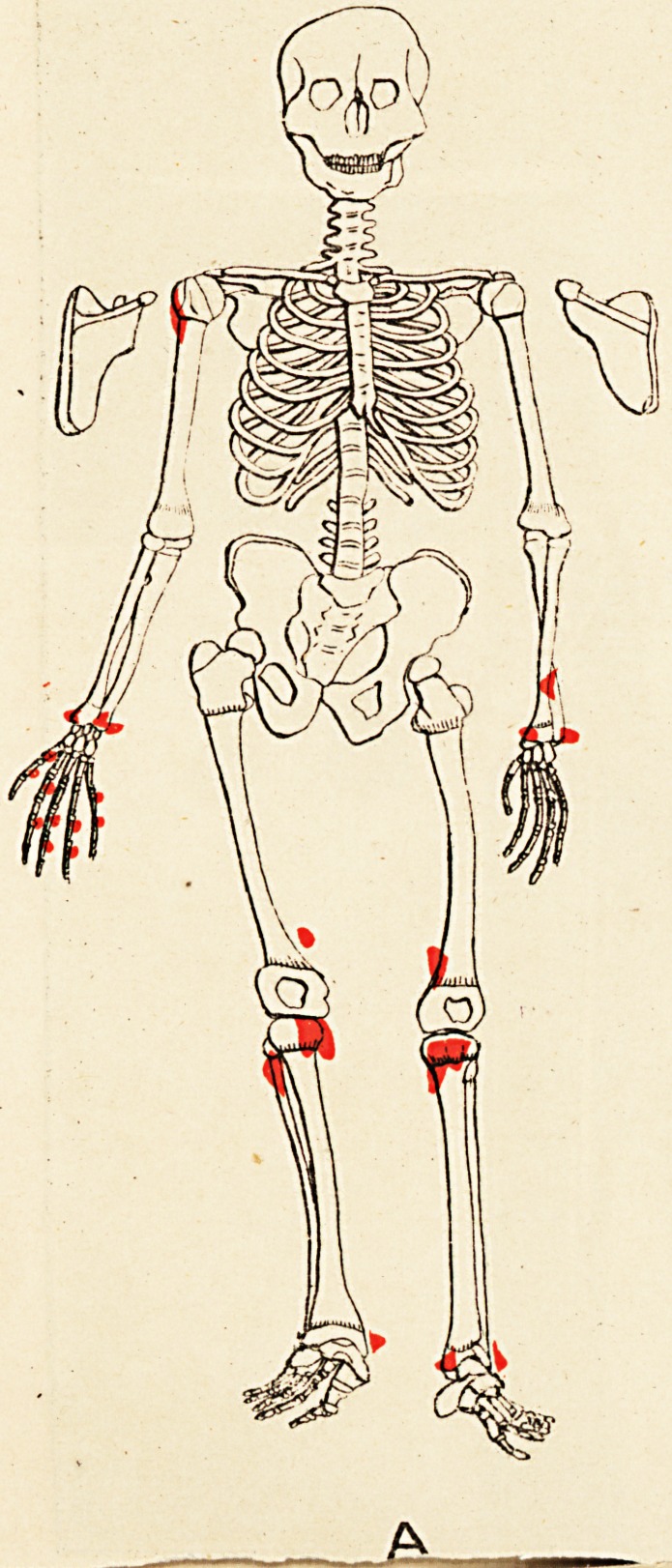


**B f3:**
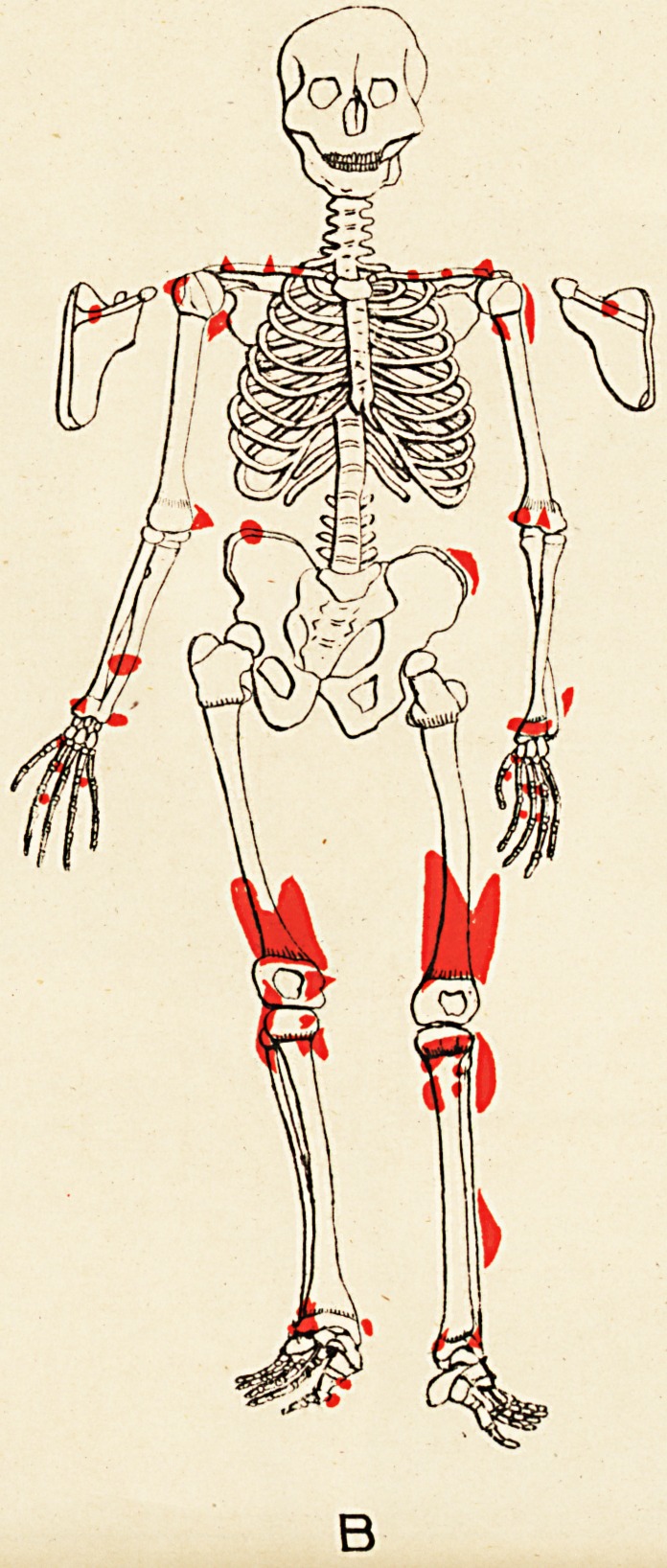


**C f4:**
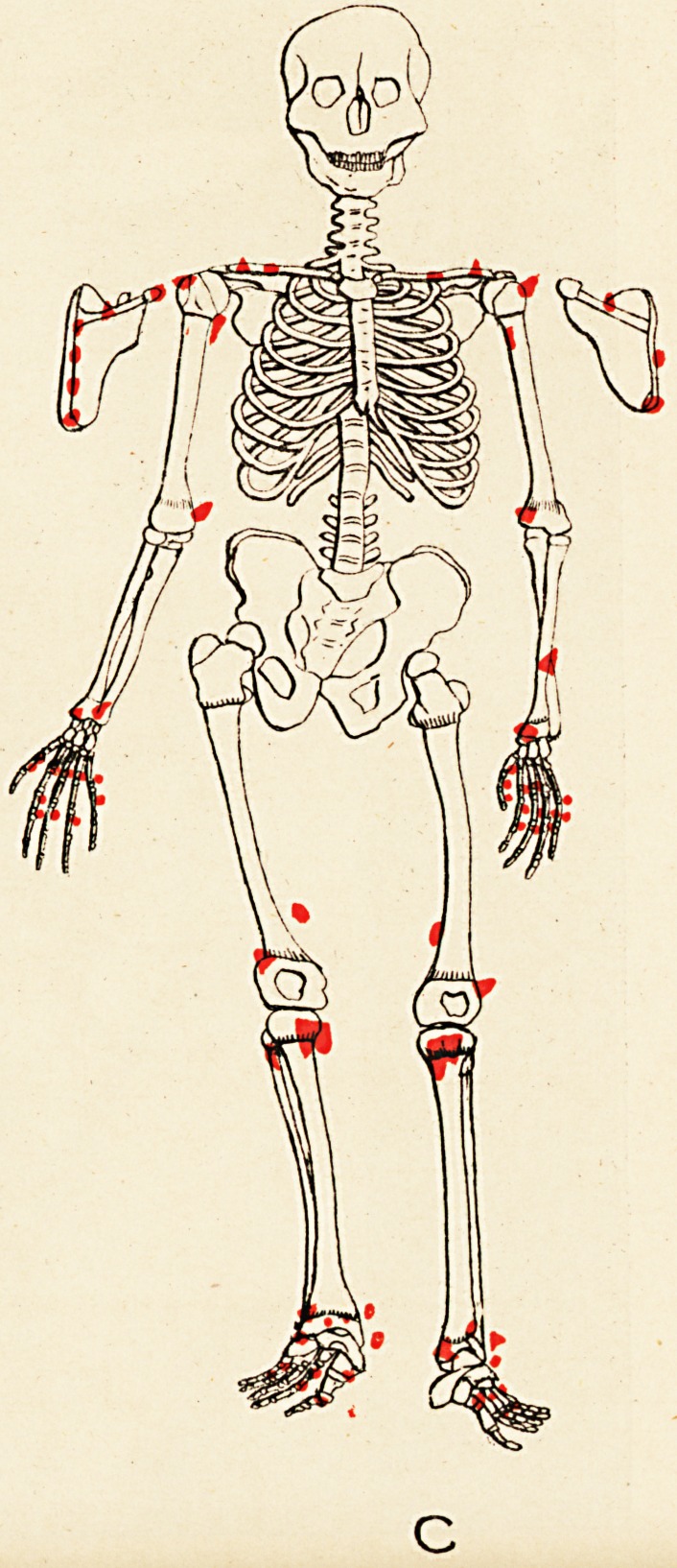


**D f5:**
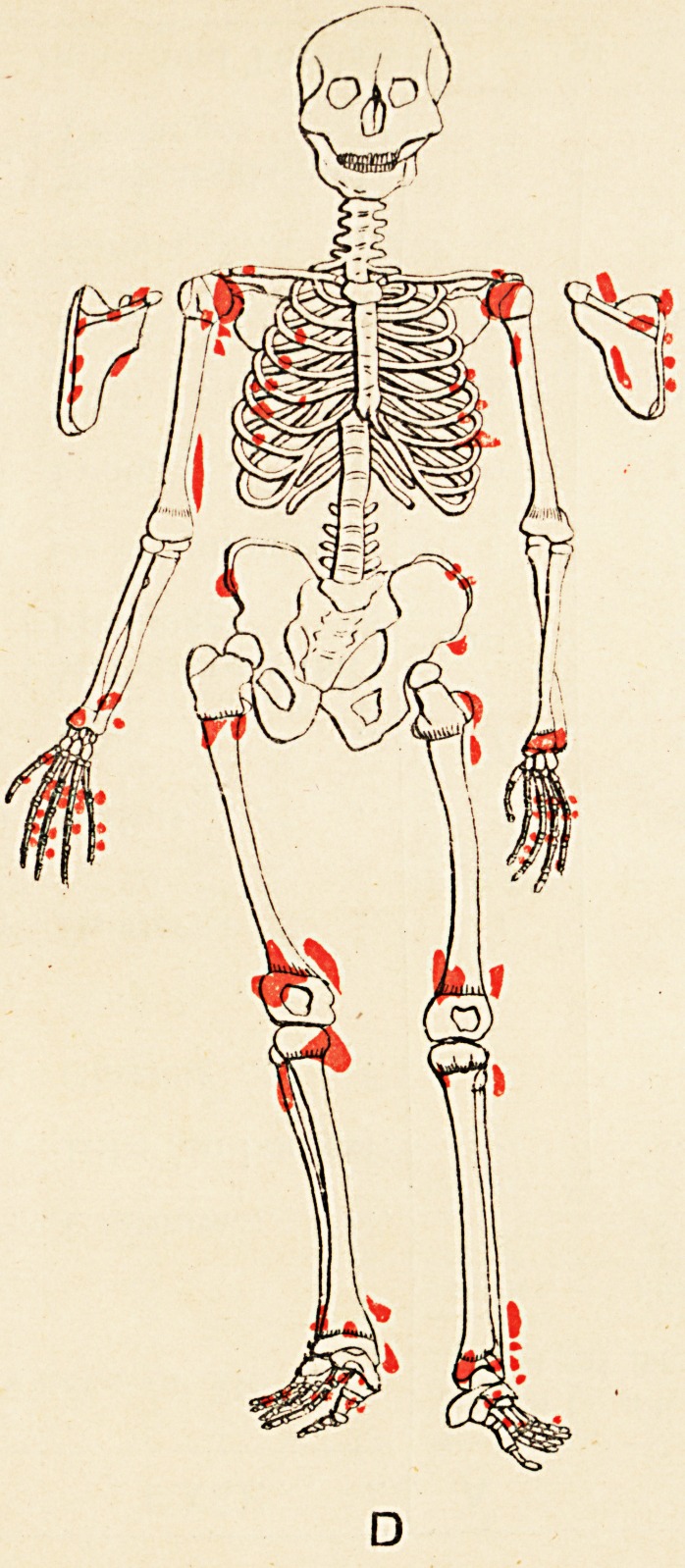


**E f6:**
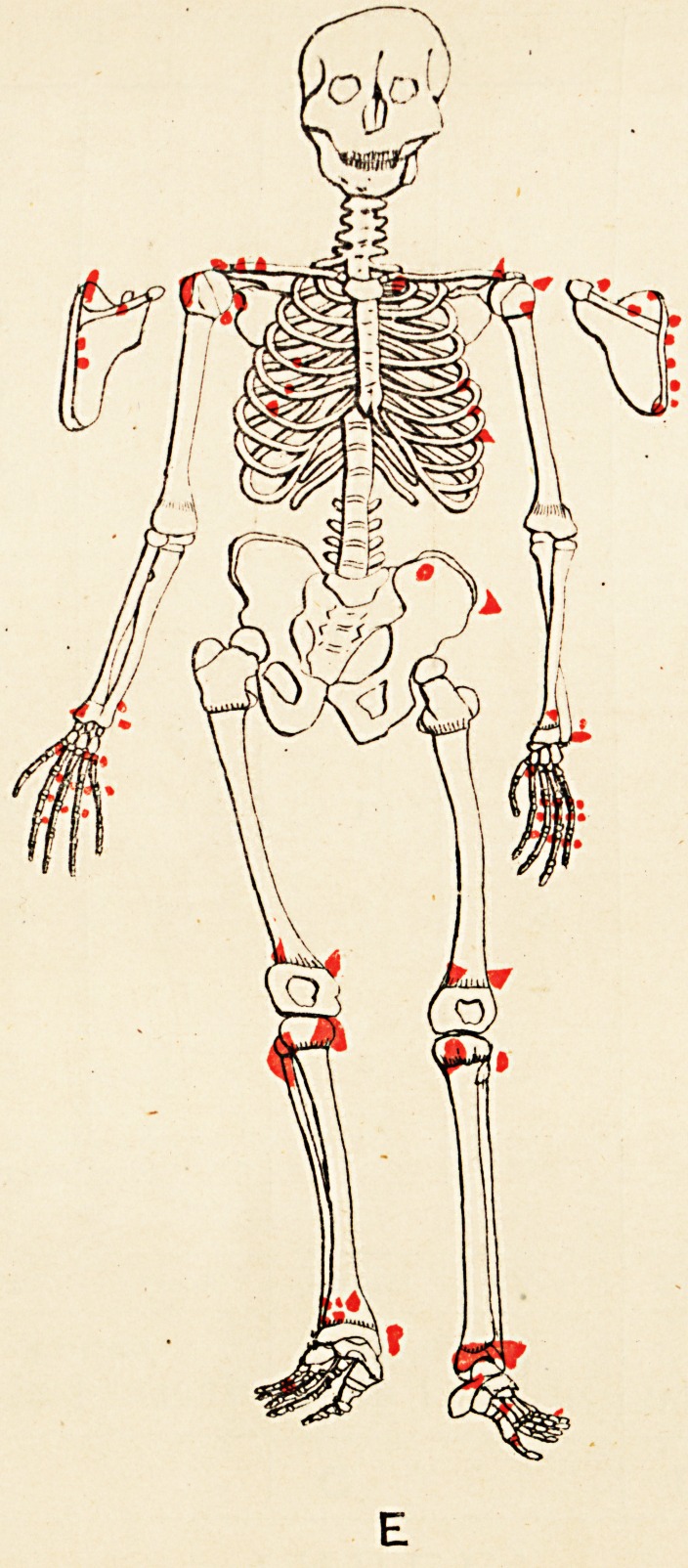


**F f7:**